# Editorial: The side effects of insecticides on insects and the adaptation mechanisms of insects to insecticides

**DOI:** 10.3389/fphys.2023.1287219

**Published:** 2023-09-22

**Authors:** Youhui Gong, Ting Li, Adil Hussain, Xiaoming Xia, Qiangli Shang, Asad Ali

**Affiliations:** ^1^ State Key Laboratory for Biology of Plant Diseases and Insect Pests, Institute of Plant Protection, Chinese Academy of Agricultural Sciences, Beijing, China; ^2^ Department of Biological Sciences, Alabama State University, Montgomery, AL, United States; ^3^ Department of Entomology, Abdul Wali Khan University Mardan, Mardan, Pakistan; ^4^ The College of Plant Protection, Shangdong Agricultural University, Taian, China; ^5^ College of Plant Science, Jilin University, Changchun, China

**Keywords:** sublethal effects, beneficial insects, insecticide resistance, insecticide resistance management, insecticide resistance mechanisms

Since their initial implementation, insecticides have played a significant role in controlling agricultural and medical insect pests. However, it is essential to recognize that while these chemicals effectively target insect pests, they can also harm beneficial insects, such as pollinators and natural predators, and have unintended impacts on non-target insects. Additionally, the use of insecticides raises concerns about their lasting impact on the environment and the delicate balance of ecosystems, affecting various organisms within these intricate systems (Ansari et al., 2014). Moreover, the global challenge of insecticide resistance and pest resurgence poses a significant constraint on agricultural output (Zuo et al., 2021; Serrão et al., 2022). Considering this, “The Side Effects of Insecticides on Insects and the Adaptation Mechanisms of Insects to Insecticides” aims to explore new perspectives regarding the potential consequences of insecticide for both pest and beneficial insects, focusing on Research Topic such as hormesis effects related to aspects like reproduction, tolerance, and insect behavior. This subject also covers the biochemical and molecular mechanisms that regulate insecticide resistance and its corresponding fitness costs in insects.

Following their initial application in the field, the potency of insecticides in terms of lethal concentrations diminishes gradually, leading to exposure to low lethal and/or sublethal concentrations (Desneux et al., 2005; Desneux et al., 2007). This exposure can give rise to various sublethal effects within the affected insects, as indicated by Desneux et al. (2007). These effects encompass a range of negative impacts on crucial life history traits of beneficial insects. Afza et al. delve into the realm of sublethal and transgenerational repercussions stemming from the application of six distinct synthetic insecticides on the seven spotted lady beetle, *Coccinella septempunctata*—a valuable predatory insect. Their findings reveal that even at a low concentration (LC_30_), all tested insecticides substantially hinder the emergence of adults, adult weight, fertility, and fecundity in the parental generation when compared to the control treatment. Additionally, exposure to sublethal concentrations of these insecticides resulted in a reduction of predation rates among the F_1_ generation adults. This serves as a compelling example, highlighting the detrimental consequences of insecticides on beneficial arthropods within agroecosystems. Understanding the unintended impacts of insecticides on non-target organisms, particularly biological control agents, is imperative for the successful integration of insecticides into pest management strategies (Stark et al., 2007; Liu et al., 2019). Furthermore, this Research Topic presents yet another illustration of the consequences of insecticides on pollinators. Han et al. study uncovered that exposure to field-realistic concentrations of acetamiprid and/or difenoconazole triggered alterations in the honeybee gut microbiome and gene expression, ultimately impacting the health of these vital pollinators.

It is widely recognized that subjecting insects to mild stress from insecticides can elicit hormetic (stimulatory) responses, which holds significant implications for insect management, as well as the ecological balance and dynamics within agroecosystems (Cutler et al., 2022). Shedding light on this phenomenon, Li et al. have presented their research outcomes concerning the influence of hormesis induced by imidacloprid on the developmental and reproductive aspects of the rose-grain aphid, *Metopolophium dirhodum*. Conducted by Liu et al., another investigation delved into the sublethal impacts of emamectin benzoate on adult diamondback moths (DBM), *Plutella xylostella*, along with their subsequent generations. The most intriguing revelation from this study lies in the observation of reproductive hormesis among DBM adults when exposed to LC_20_ concentrations. The discovery of hormetic effects on reproduction holds profound significance in the realm of insect pest management, as it could potentially underlie the mechanisms driving pest resurgence following applications of insecticides (Wu et al., 2019; Cutler et al., 2022). In a recent study, Gong et al. (2023) examined the transgenerational hormetic effects of nitenpyram on fitness, as well as the development of insecticide tolerance and resistance, in *Nilaparvata lugens*. The researchers put forth the hypothesis that the population outbreaks of *N. lugens*, following exposure over multiple generations to low concentrations of nitenpyram in field crops, might arise due to increased reproduction and the subsequent development of resistance.

The emergence of insecticide resistance among insects poses a significant challenge to the long-term effectiveness of insecticides, which remain a primary means of controlling both agricultural and medical pests (Gould et al., 2018; Van Leeuwen et al., 2020). Initially, conducting thorough risk assessments of insecticide resistance is paramount as part of insecticide resistance management (IRM) strategies, particularly before the widespread adoption of new insecticides (Zhang et al., 2020). Triflumezopyrim (TFM) represents a new type of mesoionic insecticide developed by Corteva Agriscience, which showed high biological activity in controlling piercing-sucking insect pests such as planthopper (Zhang et al., 2020; Wen et al., 2021). Wen et al. reported that after 21 generations of continuous selection with TFM, the *Laodelphax striatellua* developed a 26.29-fold resistance to TFM with no cross-resistance to five other insecticides. This research indicated that there is a risk of insecticide resistance development during the continuous selection of TFM in fields, which is consistent with the results obtained by Zhang et al. (2022). Furthermore, insecticide resistance monitoring within field populations across different regions holds paramount importance in IRM (Huang et al., 2021). This practice aids in making informed choices regarding insecticides and the rotation of insecticide Modes of Action (MoA) groups (Huang et al., 2021). Meng et al. meticulously assessed the susceptibility of forty-six field populations of *Chilo suppressalis* to three distinct insecticides in three provinces of Central China from 2010 to 2021. Their findings indicated that *C. suppressalis* populations developed varying degrees of resistance over time. While demonstrating moderate to high levels of resistance to triazophos, these populations still exhibited susceptibility, and low to moderate levels of resistance to chlorpyrifos and abamectin. As a result, the application of triazophos should be suspended in efforts to control *C. suppressalis*, while the frequent use of chlorpyrifos and abamectin should be reduced across Central China. Chen et al. conducted a meticulous analysis of insecticide resistance in 11 field-collected populations of fall armyworm, *Spodoptera frugiperda*, originating from Sichuan Province. The findings revealed that the resistance levels of the *S. frugiperda* field populations within Sichuan remained within a sensitive range concerning emamectin benzoate and chlorpyrifos. Notably, these populations demonstrated low to moderate resistance to chlorantraniliprole.

The development of insect resistance to insecticides can be attributed to two primary mechanisms: One mechanism involves target insensitivity resulting from gene mutations, while the other involves metabolic resistance stemming from alterations in the quantity and quality of detoxification enzyme genes (Bass and Nauen, 2023). The extensive use of diamide insecticides has notably led to the emergence of resistance among lepidopteran pests (Boaventura et al., 2020). Specifically, the G4946E and I4790M/K mutations within the ryanodine receptor (RyR) have been closely associated with resistance to diamide insecticides in various field populations of *P*. *xylostella*, as well as several other pest insects (Roditakis et al., 2017; Wei et al., 2019). Ren et al. identified two potential mutation loci, namely Gly4911Glu and Ile4754Met, that contribute to the development of insecticide resistance in the leaf beetle, *Galeruca daurica*. This beetle has emerged as a significant grassland pest, marked by sudden outbreaks in the Inner Mongolia grasslands since 2009. Remarkably, the G4911E mutant model exhibited reduced affinity and an altered mode of action towards two diamide insecticides. Consequently, their study strongly suggests that the G4911E mutation in GdRyR could potentially serve as a key mechanism driving resistance to diamide insecticides in *G. daurica*. Additional contributions within this Research Topic center on the pivotal role of detoxification enzymes and genes in the development of insecticide resistance. Wen et al. have proposed that the development of Triflumezopyrim resistance in *L. striatellus* is closely linked to several P450 genes. In the 12th paper, Chen et al. have illuminated the upregulation of five specific P450 genes (*CYP6AE43*, *CYP321A8*, *CYP305A1*, *CYP49A1*, and *CYP306A1*) as potential contributors to the moderate-level resistance observed towards chlorantraniliprole. Furthermore, the eighth paper conducted by Chen et al. have confirmed the critical role of a cytochrome P450 gene, MusiDN2722 in the resistance of *Megalurothrips usitatus* Bagnall to acetamiprid through RNA interference targeting the P450 gene.

The phenomenon of insecticide resistance often carries a fitness cost, forming the foundation for resistance management strategies that involve altering insecticide usage (ffrench-Constant and Bass, 2017). However, this principle does not hold in every scenario. Hasnain et al. have presented findings indicating that the chlorantraniliprole-reduced susceptible strain of *S*. *frugiperda* demonstrates higher performance in fecundity and other life table traits compared to the chlorantraniliprole-susceptible strain. To counteract the emergence of insecticide resistance, it is imperative to implement a multifaceted approach encompassing biological control, crop rotation, transgenic plants, and cultural practices, in conjunction with refined insecticide application strategies. These refined insecticide application strategies include alternating insecticide usage, adjusting mixtures, and reducing application frequencies. Feng et al. introduced an innovative approach wherein combining pymetrozine with the fungicide zhongshengmycin amplifies the insecticidal effects of pymetrozine while concurrently carrying fitness costs in *Nilaparvata lugens* (Stål), which has developed elevated resistance to pymetrozine. This research unveils a fresh avenue to stave off resistance development and enhance the efficacy of pest management. In another exploration, Idrees et al. evaluated the insecticidal potency of the entomopathogenic fungus *Metarhizium anisopliae* MA against *S*. *frugiperda*. Their study demonstrated the efficacy of this fungus in inducing mortality among second instars, eggs, and neonate larvae under controlled laboratory conditions.

In summary, this Research Topic delved into the multifaceted aspects of insecticide’s impact on insects and their adaptive responses. The outcomes illuminated various dimensions, encompassing effects on pollinators and predators, hormesis effects influencing pest insect populations, risk assessment for insecticide resistance, and the intricate mechanisms underlying the development of insecticide resistance. Furthermore, valuable instances of utilizing biological control and combining pesticides with fungicides for pest management and resistance mitigation were highlighted within this Research Topic. However, despite these significant advancements, numerous questions remain unanswered. For instance, while many studies have identified an array of detoxification genes exhibiting overexpression in resistant insects, the exact roles of these genes require further investigation and validation in the future.

We extend our gratitude to all authors for their insightful contributions, as well as to the reviewers and the dedicated editorial team for their invaluable input, comments, and suggestions. With its diverse insights, we anticipate that this Research Topic will prove engaging and enlightening for the broad readership of Frontiers in Physiology.
